# Human immunodeficiency virus, tuberculosis and malaria service readiness at the primary healthcare centers in Ekiti State, Nigeria

**DOI:** 10.11604/pamj.2022.43.116.35883

**Published:** 2022-11-01

**Authors:** Adeyinka Adeniran, Chisom Florence Chieme, Omobola Yetunde Ojo, Esther Oluwole, Babatunde Olujobi, Marcus Ilesanmi

**Affiliations:** 1 Lagos State University College of Medicine Nigeria, Lagos, Nigeria,; 2 Petra Global Consulting, 71 Akanro Road Ilasa Lagos State, Lagos, Nigeria,; 3 Federal Medical Center Abeokuta, Ogun State, Abeokuta, Nigeria,; 4 College of Medicine, University of Lagos, Lagos, Nigeria,; 5 State Primary Health Care Development Agency, Ado Ekiti, Ekiti State, Nigeria,; 6 College of Medicine, University of Saskatchewan, Saskatoon, Canada

**Keywords:** Service readiness, health facilities, access, quality, availability

## Abstract

**Introduction:**

access to services encompasses three components: availability, affordability, and acceptability. The physical presence of service delivery, which includes health infrastructure, core health staff, and aspects of service use, is referred to as service availability. This study was conducted to inform the health service availability and preparedness to deliver HIV, TB, and malaria prevention and control services in Ekiti State.

**Methods:**

this is a descriptive cross-sectional study conducted among all the Primary Health Centres (177) in Ekiti State Nigeria between August and October 2020. Data were collected with the use of the World Health Organization Service Availability and Readiness Assessment tool and were analyzed using STATA SE 12.

**Results:**

close to half (49%) of them had a condom in supply. More than 90% of them provided diagnosis and treatment of malaria. The HIV-specific service readiness index was approximately 40/0%. Only 26.6% of health facilities were ready to offer TB prevention and control services. Malaria specific service readiness index was 61.9%. There was a statistically significant difference in the HIV and TB-specific service readiness of facilities in the urban compared to rural locations. Health facilities located in the urban areas had higher mean readiness scores compared to those in the other residential areas (P=0.014).

**Conclusion:**

it is evident that HIV and TB-specific service readiness is very poor among PHCs in Ekiti State. Malaria Service Readiness was fair. Ekiti State government needs to expand investments in PHCs by strengthening the diagnostic services, commodities and medicine supply, adequate equipment and staff training.

## Introduction

All over the world, countries are committed to ensuring that quality health services are accessible to all, without financial burden by 2030, as part of the Sustainable Development Goals (SDG) of 2015. Successful primary healthcare (PHC) is pivotal to achieving this aim [1]. PHC offers a wide range of services to the public, including both curative and preventive care. The primary healthcare centres are important avenues for low-and middle-income countries to gain access to PHC services [2]. One of the key roles of a health system is to ensure that people have access to quality healthcare. Access to services encompasses three components: availability, affordability, and acceptability. The physical presence of service delivery, which includes health infrastructure, core health staff, and aspects of service use, is referred to as service availability. The availability of components needed to provide services, such as basic facilities, basic equipment, standard infection prevention precautions, diagnostic capability, and necessary medicines, is referred to as service readiness [2]. Quality service is also essentially required for service readiness. In accomplishing the health-related SDGs, it is pertinent to re-evaluate the preparedness of healthcare facilities for service delivery. The state of service delivery in Nigeria's health sector has been a source of constant criticism for decades [3].

Human Immunodeficiency Virus (HIV), Tuberculosis (TB), and malaria are a huge part of the global burden of diseases. The 2015 report on the global burden of diseases had shown that HIV and Acquired Immune Deficiency Syndrome (AIDS) caused 1.2 million deaths [4]. In Nigeria 1.8 million people were living with HIV as of 2019. Women were the most afflicted, accounting for around one million people. There were 150,000 HIV-positive children under the age of 14 [5]. Nigeria is the world's third-largest of HIV-infected country [6], HIV continues to be a global public health problem, with targets of 90-90-90 by 2020 and AIDS eradication by 2030. Owing to the inadequate coverage of the prevention of mother-to-child transmission (PMTCT) program, Nigeria has one of the highest rates of vertical HIV transmission in the world. While anti-retroviral (ARVs) are still mostly provided at the tertiary and secondary levels of care, a major initiative to decentralize ARV service delivery to primary and community health centres began in 2011. Sadly, access to HIV care continues to be a challenge, particularly for the urban poor and rural communities, where clients mainly receive care at the primary healthcare facilities [4]. TB caused 1.1 million deaths while malaria and neglected tropical diseases caused 843.1,000 deaths [4]. A study conducted in Nepal, reported that the average service readiness score for HIV therapy and testing was 68.9% [7]. Concerning TB, Nigeria was ranked 1^st^ in Africa and 6^th^ among the 30 countries with the highest burden in the world [8]. Nigeria is one of the 14 countries on the World Health Organization's (WHO) global high-burden country with an estimated incidence rate of 219 per 100,000 people and a mortality rate (excluding HIV-positive people) of 64/100,000 [8]. Effective preventive measures, early detection, and adequate treatment could help reduce the burdens of tuberculosis. However, if health facilities are widely available and ready to provide TB treatment, this may be accomplished. One of the fundamental functions of a health system is to ensure access to quality healthcare services, but due to poverty, most developing counties have failed to do so [9]. As indicated by the most recent world malaria report that was released in the year 2020, there were 229 million malaria cases and 409,000 malaria deaths in 2019.

Nigeria accounted for 23% of all malaria deaths around the world. Malaria can be controlled and deaths can be prevented with early diagnosis and treatment. It also aids in the reduction of the spread [10]. A study on hospital readiness for inpatient malaria case management in 22 Nigerian hospitals found that nearly three-quarters (73%) offered malaria microscopy, 27% stocked non-expired RDTs, and 23% of hospital laboratories were unable to provide at least one parasitological malaria diagnosis despite RDTs complementing diagnostic services [11]. A recently concluded survey reported that the higher the service readiness index, the lower the mortality rate in peripheral health centres in Burkina Faso [12]. There are variances in the quality of services provided by private and public providers, as well as regional differences in Nigeria. In certain cases, Nigeria's healthcare scheme has been found to function underneath benchmarks in terms of the availability of human resources, essential infrastructure, equipment, and medications [3]. Hence, the need to scale up the service readiness and availability in health facilities. This study was conducted to assess the health service availability and preparedness to deliver HIV, TB, and malaria prevention and control services at the primary healthcare level in Ekiti State. The findings will be crucial in identifying gaps that can be used to drive resource allocation and service delivery in the State.

## Methods

**Study design:** this was a descriptive cross-sectional study.

**Study setting:** Ekiti State, with its capital in Ado-Ekiti, is one of the thirty-six States that make up Nigeria. It was created on October 1^st^, 1996. According to the 2016 estimate, it has a population of 3,270,798 people on a landmass of 5,435 km and a 2.3% annual growth rate [13,14]. The State is predominantly agrarian, with urban and semi-urban areas comprising small and medium-sized businesses. The State consists of 326 PHC facilities with a staff capacity of 4,261 at the time of the study [15]. Ekiti State has 16 Local Government Areas (LGAs) and 6 federal constituencies; Ekiti Central 1 (Ado, Irepodun, Ifelodun), Ekiti Central II (Ijero, Efon, Ekiti West), Ekiti North I (Ikole, Oye), Ekiti North II (Ido-Osi, Ilejemeje, Moba), Ekiti South I (Ekiti South-west, Ikere, Ise-Orun), Ekiti South II (Ekiti East, Emure, Gbonyin). Each LGA has a minimum of ten political wards and a total of 177 political wards in the State [16].

The components of the WHO's Service Availability and Readiness Assessment (SARA) tool were adapted to focus mainly on HIV, TB and malaria service-specific readiness and availability were used for the collection of data [17]. Four domains were measured for each of the facilities; basic equipment, diagnostics, staff training and guideline, medicine and commodities [17]. The survey was statewide and the tool was administered by sixteen trained research assistants who were healthcare workers with at least a post-secondary school certificate. One-day intensive training was conducted from 9:00am to 6:00pm. The questionnaire was interviewer-administered using kobocollect tool (an open-source Android app for survey data collection). Data collection took place between August and October 2020. The questionnaire was pretested in similar semi-urban and rural PHCs facilities in Lagos State for corrections before the commencement of the study.

**Study participants:** the study was conducted among the administrative heads (Officer in Charge) of Primary Health Centres and Primary Health Clinics in Ekiti State, Nigeria with the exclusion of health posts. According to the Ward Minimum Health Care Package (WMHCP) which was developed to address the current strategy to deliver PHC services: based on the Ward Health System, the three recognized facility types are; Health Post, Primary Health Clinic and Primary Health Care Centres [18]. The officers who had been in charge of the facilities for at least six months were respondents in this study.

**Study size:** the minimum sample size was determined using Cochrane's formula for finite numbers [19] to be 172 with 95% confidence interval and 62% estimated proportion of the general service readiness index for Kaposia and Screepur Upazila [19]. The total sampling of all PHC facilities was done for better representation of the State.

### Variables

***Outcomes measures:*** overall specific service readiness for HIV, TB and Malaria respectively. Domain Specific (basic equipment, diagnostics, staff training and guideline, medicine and commodities) service readiness for HIV, TB and Malaria respectively.

***Explanatory variables:*** type of facility, urban/rural and federal constituency.

**Operational Definitions of Terms**

***Service availability:*** this is the physical presence of service delivery, it also includes; health infrastructure, core health personnel and aspects of service utilization [17].

***Service readiness:*** this refers to the availability of components required to provide services like: basic equipment, basic amenities, standard precaution for infection prevention, diagnostic capacity and essential medicines [17].

**Data sources/measurement:** SARA tool was used for data collection. Four domains were measured for each of the facility; basic equipment, diagnostics, staff training and guideline, medicine and commodities [17].

**Bias:** the research assistants were thoroughly trained on the instruments to prevent information bias. Also, an observer checklist was used by the research assistants to prevent social-desirability bias. It is an objective checklist which is a component of the SARA tool, an instrument used to physically confirm the availability of equipment in the Primary Healthcare facilities.

**Quantitative variables:** domain score (basic equipment, diagnostics, staff training and guideline, medicine and commodities) for each facility was carried out using the formula; n/tracer items X 100. Where n is the total number of items available in each facility and the denominator is the number of indicator tracer items for each of the domains (basic equipment, diagnostics, staff training, and guideline, medicine and commodities). The overall score for HIV, TB and malaria service readiness was derived from this formula; the average score of the domains/number of domains X100.

**Data management:** the completed questionnaires from kobocollect were cleaned and coded on Microsoft Excel 2016 and exported to STATA SE 12 (STATA CORP LLC, College Station, Texas, USA) for analysis. A score of “1” was awarded when a relevant item required for service delivery was available and “0” mark awarded when it was not available. Percentage and frequency distribution were used to present the various HIV, TB and malaria services available at PHC facilities. Domain score (basic equipment, diagnostics, staff training and guideline, medicine and commodities) for each facility was carried out using the formula; n/tracer items X100. Where n is the total number of items available in each facility and the denominator is the number of indicator tracer items for each of the domains (basic equipment, diagnostics, staff training, and guideline, medicine and commodities). The overall score for HIV, TB and malaria service readiness was derived from this formula; the average score of the domains/number of domains X100 [17]. The results of the categorical variables were presented as frequencies and percentages while ANOVA and independent t-test was used to assess the association between the outcome (HIV, TB, and Malaria specific service readiness respectively) and independent variables such as facility type, urban/rural areas, and federal constituency. The State and local government area headquarters were referred to as urban areas and vice versa. The level of significance was set at a p-value less than 0.05.

**Ethical approval:** ethical approval for the study was obtained from the Health Research and Ethics committee of Lagos State University Teaching Hospital (REF. N° - LREC/06/10/1424). Written informed consent was obtained from each respondent with assurance of confidentiality of information and their right to withdraw from the study at any point in time. They were made to understand that involvement was voluntary and had nothing to do with their employer.

## Results

**Distribution of healthcare facilities in Ekiti State:** the majority (81.9%) of the health facilities were located in rural areas. About 18.6% were located in Ekiti Central II constituency and 94.9% were Primary Health Centres (Table 1).

**Table 1:** distribution of healthcare facilities

Variable	N=177 n (%)
**PHC location**	
Rural	145 (81.9)
Urban	32 (18.1)
**Federal constituency**	
Ekiti North I	23 (13.0)
Ekiti North II	32 (18.1)
Ekiti South I	32 (18.1)
Ekiti South II	32 (18.1)
Ekiti Central I	25 (14.1)
Ekiti Central II	33 (18.6)
**Type of facility**	
Primary health clinic	168 (94.9)
Health clinic	9 (5.1)

**HIV, TB and malaria specific-service readiness at the primary healthcare facilities:** one-third (33.3%) of health facilities had the national HIV counselling and testing guideline. close to half (49.2%) of them had a condom in the facilities. Only 13.6% had the national guidelines for TB clinical management and 15.8% offer TB infection control. About 54.8% had the national guidelines on malaria treatment and diagnosis, but more than 90.0% of the health facilities provide diagnosis and treatment of malaria (Table 2).

**Table 2:** HIV, TB, and malaria specific service readiness at the primary healthcare facilities

HIV, TB and malaria service readiness (N=177)	n (%)
**HIV service readiness**	
Availability of National ART guidelines	
Availability of National HIV counselling and testing guidelines	59 (33.3)
Training of staff on involuntary counselling and testing	36 (20.3)
Training on HIV prevention care and management	46 (26.0)
Training on ART prescription	10 (5.7)
HIV counselling and testing services	109 (61.6)
Availability of private room/area for HIV testing and counselling services	76 (42.9)
Availability of condoms	87 (49.2)
**TB service readiness**	
National guidelines for TB clinical management	24 (13.6)
Offers TB diagnostics services	28 (15.8)
TB diagnosis by microscopy	16 (9.0)
TB diagnosis by culture	8 (4.5)
TB diagnosis by rapid test MTB/RIF	11 (6.2)
TB diagnosis by chest x-ray	6 (3.4)
Offer drug prescription for TB patient	23 (13.0)
Management and treatment follow-up for TB patients	33 (18.6)
Test TB patients for HIV	18 (11.5)
Management of HIV and TB co-infection	19 (10.7)
MDR-TB	11 (6.2)
TB infection control	28 (15.8)
Provide preventive treatment for TB (INH + Pyrido)	18 (10.2)
Availability of Ethambutol	12 (6.8)
Availability of Isoniazid + Rifampicin (2FDC)	13 (7.3)
Availability of Isoniazid + Rifampicin + Pyrazinamide + Ethambutol (4FDC)	15 (8.5)
**Malaria service readiness**	
Availability of national guidelines on malaria treatment and diagnosis	97 (54.8)
Malaria diagnosis	166 (93.8)
Malaria diagnosis by clinical symptoms	141 (79.7)
Malaria diagnosis by RDT	148 (83.6)
Malaria diagnosis by microscopy	22 (12.4)
Malaria treatment	164 (92.7)
Intermittent preventive treatment for malaria	117 (66.1)

**Domain-specific HIV, TB and malaria service readiness index at the PHC facilities:** HIV-specific service readiness index was 39.7%. Only 26.6% of health facilities were ready to offer TB services while Malaria specific readiness index was 62.0% (Table 3).

**Table 3:** domain-specific HIV, TB and malaria service readiness index at the PHC facilities

Domain specific readiness (n=177)	Index (mean percent)
**HIV specific service readiness**	39.7
Guidelines and training availability	21.0
Diagnostic services availability	48.6
Equipment availability	73.3
Medicine and commodities availability	15.9
**TB specific service**	26.6
Diagnostic services availability	11.1
Medicine and commodities availability	12.1
Guidelines and training availability	10.0
Equipment availability	73.3
**Malaria specific service**	62.0
Diagnostic services availability	67.4
Guidelines and training availability	43.1
Medicine and commodities availability	60.6
Availability of equipment	76.7

**Domain-specific HIV, TB, and malaria service readiness index among health facilities in the urban and rural areas:** the availability of HIV diagnostics services and malaria commodities and medicines were statistically associated with urban/rural areas. HIV diagnostic services and malaria medicines were more available in the PHCs in the urban areas compared to those in the rural areas. (P<0.05) (Table 4).

**Table 4:** domain-specific HIV, TB, and malaria service readiness index among health facilities in the urban and rural areas

HIV, TB, and malaria service readiness	Rural (Mean±SD)	Urban (Mean±SD)	/T-test/	P-value
**HIV specific readiness**				
Availability of HIV equipment	0.72±0.31	0.79±0.25	1.12	0.264
Availability of HIV diagnostics services	0.45±0.38	0.64±0.37	2.49	0.014*
Availability of HIV guidelines and training	0.19±0.25	0.29±0.28	1.84	0.067
Availability of HIV commodities and medicine	0.15±0.17	0.21±0.22	1.81	0.073
**TB specific readiness**				
Availability of TB equipment	0.72±0.31	0.79±0.25	1.12	0.264
Availability of TB diagnostics services	0.10±0.20	0.18±0.30	1.95	0.052
Availability of TB guidelines and training	0.09±0.22	0.16±0.33	1.46	0.146
Availability of TB commodities and medicine	0.11±0.20	0.19±0.29	1.82	0.070
**Malaria specific readiness**				
Availability of Malaria equipment	0.75±0.32	0.84±0.27	1.46	0.147
Availability of Malaria diagnostics services	0.68±0.23	0.66±0.15	0.28	0.779
Availability of Malaria guidelines and training	0.43±0.40	0.46±0.35	0.43	0.666
Availability of Malaria commodities and medicine	0.59±0.22	0.69±0.19	2.48	0.014*

**HIV, TB, and malaria service-specific readiness indices across different healthcare facility types, urban/rural, LGA:** HIV and TB-specific readiness was statistically significant to the federal constituency and urban/rural areas. Health facilities located in the urban areas and Ekiti North 1 had higher mean readiness scores. There were statistically significant differences between malaria-specific readiness and federal constituencies (p<0.05) (Table 5).

**Table 5:** HIV, TB, and malaria service-specific readiness indices across different healthcare facility types, urban/rural, LGA

Variable N=177	HIV Specific Readiness Mean(SD)	F-Ratio /t-test/	P-value	TB Specific Readiness Mean(SD)	F-Ratio /t-test/	P-value	Malaria Specific Readiness Mean(SD)	F-Ratio /t-test/	P-value
**Type of Facility**									
Primary health clinic	0.41±0.22	3.68	0.057	0.27± 0.18	1.83	0.178	0.62±2.00	1.95	0.164
Health clinic	0.26±0.20			0.19± 0.17			0.53±0.18		
**PHC location**									
Rural	0.38±0.22	5.74	0.018*	0.25± 0.17	4.46	0.036*	0.61±0.21	1.96	0.163
Urban	0.48±0.21			0.33± 0.22			0.66±0.14		
**Federal constituency**									
Ekiti North I	0.52±0.26	3.48	0.005*	0.37± 0.24	2.94	0.014*	0.81±0.18	0.85	<0.001*
Ekiti North II	0.38±0.20			0.24±0.17			0.60±0.18		
Ekiti South I	0.30±0.23			0.21±0.18			0.50±0.21		
Ekiti South II	0.45±0.17			0.29±0.11			0.66±0.12		
Ekiti CentralI	0.37±0.19			0.30±0.19			0.60±0.15		
Ekiti Central II	0.39±0.22			0.22±0.18			0.61±0.20		

* P-value < 0.05 at 5% significance level. ^/ttest/

## Discussion

The outcome of this study determined the need for the provision of quality HIV, TB, and malaria-specific services in the primary healthcare facilities located in both rural and urban areas in Ekiti State. HIV-specific service readiness index in this study was nearly 40.0%. This finding is lower compared to a study conducted in Nepal 68.9% [7], and this could be attributed to the fact that the study was conducted in both private and public owned facilities. This study found that 48.6% offer HIV diagnostic services and approximately 16.0% provide medicine and commodities to HIV-positive patients. This is lower than the outcome of a study conducted in Enugu State, which reported that among 68.0% of PHCs offer HIV testing services, only 25.0% offered treatment [2]. The variation might be due to difference in study population. The present study found that 42.9% of the PHCs offer both visual and auditory services, condoms were available in 49.2% of the health facilities, about 26.0 % of staff were trained on HIV prevention and management and only 5.1% have the ART national guidelines in their facilities. These findings are different from that of a study conducted in Nepal (96.0%, 62.0%, 38.8%, 34.0% respectively) [7]. Health workers need to be trained on proper management of HIV because PLWHA need to maintain good health while they are on ART and consequently require viable preventive administrations. When they start, they will require clinical care to remain healthy and avoid side effects [20].

The present study found that the TB service readiness score was the lowest compared to HIV and malaria. Only 26.6% of health facilities were ready to administer TB services. In contrast to other studies which reported 53.0% in Mongolia [21], 73.0% in Ethiopia [22], 76.5% in Oyo, and 73.6% in Anambra [23]. Hence the deficiency in TB service readiness in the present study might be due to inadequate TB diagnostic services (11.1%), medicines and commodities (12.1%), and national guideline/staff training (10.0%). Lack of staff training on TB care is related to noncompliance with the national treatment standards. Inadequate TB management gives rise to potential transmission dangers, poor prognosis, development of drug-resistant TB, and improper treatment. The Xpert MTB/RIF has been proposed by WHO in place of sputum microscopy since it is quicker, more reliable, and correct. Poor diagnostic services delay the commencement of treatment and increase the spread of the infection [23]. This study found that only 11.5% offer diagnosis of TB among HIV-positive patients. However, a previous study conducted in Nigeria reported that all the health facilities undergo HIV diagnosis among TB patients. The outcome variation may be due to differences in the study population, the previous study was conducted among primary, secondary, and tertiary DOTS facilities [23].

In this study, 62.0% of health facilities were ready to deliver malaria services. This finding was higher than that of a study conducted in Ethiopia which reported that 52.0% of health facilities were ready to conduct malaria services [24]. This study found that close to 70.0% offered malaria diagnostic services. This finding was higher than that of a study conducted in Somalia (57.0%) [25]. The current study found that 83.6% of health facilities diagnose malaria by RDT, 12.4% by microscopy, and approximately 80.0% by clinical symptoms. This was not similar to the findings of a study conducted in Kano which reported that 73.0% of health facilities offer malaria diagnostic services by microscopy while 27.0% by RDT. The difference could be attributed to the study being conducted in institutions that offered in-patient services [11]. Malaria service readiness is very essential in both urban and rural PHCs, Inadequate preparedness for malaria in lower-level facilities, which serve a large proportion of the rural population, could explain why the disease remains the leading cause of mortality [26].

In this study, PHCs located in the urban area were more ready to offer HIV, TB, and malaria-specific services than those in the rural area. This is similar to a study conducted in Uganda [26]. The current study found that there was a statistically significant difference between HIV and TB service readiness index and the facility location, and their federal constituency (p<0.05), but there was no association between HIV, TB, malaria and the health facility types. This finding is different from that of a study conducted in Ethiopia which reported that there was a statistically significant association between health facility types and mean score readiness [24].

Six key systematic factors for poor performance of the health systems in Nigeria were discovered to be, constrained federal government, State government, and LGA with public financing, immoderate dependency on cost recovery mechanisms, a highly fragmented governance structure, duplicate responsibilities, and undefined authorities, and poor staffing and management. All these factors reflect two comprehensive system-level barriers: financing and governance, which are the major causes of malfunction observed in the Nigerian PHC system [27].

**Limitations of this study:** this is the first study we are aware of that has used the SARA tool in the assessment of HIV, TB and malaria service readiness in PHC facilities in Ekiti State, Nigeria. The nature of cross-sectional study made it difficult to determine causality.

## Conclusion

Based on the findings from this study, it is evident that the PHCs in Ekiti State were poorly ready to provide HIV and TB services, but were quite ready to offer malaria services. Ekiti State government needs to expand investments in PHCs by strengthening the diagnostic services, training of staff, commodities and medicine supply, and provision of adequate equipment.

### What is known about this topic

- Low and middle-income countries (LMICs) still lack a well-defined structure of primary healthcare strategy to effectively provide quality HIV, TB and Malaria care services;
- Healthcare service delivery in Nigeria is frequently marred by underfunding and poor management, negatively impacting coverage and quality.

### What this study adds

- The present study revealed that the primary healthcare systems in Ekiti State Nigeria were poorly ready to provide HIV and TB services, but fairly ready to provide malaria prevention and control services;
- Bringing to fore the need for improvement in the provision of quality HIV, TB and malaria specific services and health systems strengthening.
